# Deep Learning Based on Computed Tomography Predicts Response to Chemoimmunotherapy in Lung Squamous Cell Carcinoma

**DOI:** 10.14336/AD.2024.0169

**Published:** 2025-05-12

**Authors:** Jie Peng, Baowen Xie, Honglian Ma, Rui Wang, Xiao Hu, Zhongjun Huang

**Affiliations:** ^1^Department of Medical Oncology, the Second Affiliated Hospital, Guizhou Medical University, Kaili, China.; ^2^X-Lab, Seaever, Shenzhen, China.; ^3^Department of Radiation Oncology, Cancer Hospital of the University of Chinese Academy of Sciences, Hangzhou, China.; ^4^Department of Thoracic Surgery, the Third Affiliated Hospital of Chongqing Medical University, Chongqing, China

**Keywords:** deep learning, computed tomography image, major pathological response, neoadjuvant chemoimmunotherapy, lung squamous cell carcinoma

## Abstract

Non-small-cell lung carcinoma (NSCLC) often carries a dire prognosis. The advent of neoadjuvant chemoimmunotherapy (NCI) has become a promising approach in NSCLC treatment, making the identification of reliable biomarkers for major pathological response (MPR) crucial. This study aimed to devise a deep learning (DL) model to estimate the MPR to NCI in lung squamous cell carcinoma (LUSC) patients and uncover its biological mechanism. We enrolled a cohort of 309 LUSC patients from various medical institutions. A ResNet50 model, trained on contrast-enhanced computed tomography images, was developed, and validated to predict MPR. We examined somatic mutations, genomic data, tumor-infiltrating immune cells, and intra-tumor microorganisms. Post-treatment, 149 (48.22%) patients exhibited MPR. The DL model demonstrated excellent predictive accuracy, evidenced by an area under the receiver operating characteristic curve (AUC) of 0.95 (95% CI: 0.98-1.00) and 0.90 (95% CI: 0.81-0.98) in the first and second validation sets, respectively. Multivariate logistic regression analysis identified the DL model score (low vs. high) as an independent predictor of MPR. The prediction of MPR (P-MPR) correlated with mutations in four genes, as well as gene ontology and pathways tied to immune response and antigen processing and presentation. Analysis also highlighted diversity in immune cells within the tumor microenvironment and in peripheral blood. Moreover, the presence of four distinct bacteria varied among intra-tumor microorganisms. Our DL model proved highly effective in predicting MPR in LUSC patients undergoing NCI, significantly advancing our understanding of the biological mechanisms involved.

## INTRODUCTION

Among patients diagnosed with early and locally advanced non-small-cell lung cancer (NSCLC), a notable subset (roughly 20%-25%) may qualify for surgical intervention [1]. However, even after undergoing comprehensive surgical resection, a significant fraction of these patients (30%-55%) encounter postoperative relapse and ultimately succumb to their illness [2, 3]. Additionally, the integration of neoadjuvant or adjuvant chemotherapy into treatment protocols offers limited improvement in reducing recurrence, contributing only a 5% improvement to overall survival (OS). This highlights the critical demand for innovative therapeutic strategies in the treatment of resectable NSCLC [4]. In recent developments, the synergistic application of platinum-based chemotherapy with immunotherapy as a neoadjuvant chemoimmunotherapy (NCI) regimen has been introduced for NSCLC management. Early results from phase 2 trials have shown impressive major pathological response (MPR) rates, varying from 57% to 83% [5, 6], signaling a promising frontier in the battle against this challenging disease.

The CheckMate-816 clinical trial underscored the effectiveness of neoadjuvant nivolumab (Nivo) combined with chemotherapy in improving event-free survival. This breakthrough facilitated the American Food and Drug Administration's endorsement of NCI, specifically pairing Nivo with chemotherapy, as a novel standard of care for patients with resectable NSCLC [7]. Early findings from the NADIM study revealed a pronounced enhancement in 1-year progression-free survival (PFS) among patients who achieved a major pathological response (MPR) compared to those treated with NCI [8]. The association between pathological response and clinical outcomes was strikingly strong, positioning MPR as a potential predictive marker of treatment success and surrogate endpoint for evaluating long-term benefits in patients eligible for surgical intervention in NSCLC [9]. However, it's critical to acknowledge that MPR is attainable by only a subset of patients undergoing neoadjuvant immunotherapy, underscoring the imperative for a reliable biomarker to identify those most likely to benefit from NCI in the management of resectable NSCLC.

In the realm of oncology, deep learning (DL) methodologies have seen widespread application in the analysis of medical imaging and genomic sequences, playing a pivotal role in the diagnosis, prognostication, and prediction of treatment outcomes across a spectrum of cancers [10-15]. Previous studies have ventured into developing radiomics models through machine learning techniques to forecast critical indicators like programmed death-ligand 1 (PD-L1) expression, tumor mutational burden (TMB), and responsiveness to immunotherapy. These studies have yielded promising outcomes, underscoring the potential efficacy of machine learning-based methodologies [16-18]. However, the deployment of DL models based on computed tomography (CT) imaging to predict MPR in lung squamous cell carcinoma (LUSC) remains scarcely documented, leaving its potential utility in question. Furthermore, DL models often utilize an opaque, "black box" design, complicating the interpretation of their predictions, a significant hurdle in medical contexts. Thus, the exploration of DL models for predicting MPR to NCI in patients with LUSC, particularly in elucidating the intricacies of DNA and RNA sequence data, presents a largely untapped opportunity.

In this study, we harnessed the power of the ResNet 50 algorithm, utilizing contrast-enhanced CT (CE-CT) images, to create a sophisticated model. This model is designed to categorize individuals with LUSC into those likely to achieve MPR and those who might not (No-MPR) following NCI and before surgical intervention. We evaluated the predictive performance of our DL model, comparing it against traditional radiological assessments in forecasting MPR through multivariate analyses. Upon refining our DL model, we analyzed the CE-CT images corresponding to predicted MPR (P-MPR) outcomes, employing the CAM-Grad method to highlight significant regions of interest. Additionally, we undertook a comprehensive investigation that spanned somatic mutations, genomics, and the spectrum of immune cells within the tumor microenvironment (TME) and circulating in peripheral blood, as well as the diversity of intra-tumor microorganisms, to discern the distinctions between the MPR and No-MPR groups. Our goal was to deliver a powerful and non-invasive predictive tool that could empower oncologists to devise more tailored NCI approaches for patients with LUSC.

## MATERIAL AND METHODS

### Patients

Patients undergoing NCI at three distinct medical centers—the Cancer Hospital of the University of Chinese Academy of Sciences, the Third Affiliated Hospital of Chongqing Medical University, and the Second Affiliated Hospital of Guizhou Medical University—were allocated into a discovery cohort (n = 200), a first validation cohort (n = 60), and a second validation cohort (n = 49). Additionally, DNA and RNA sequencing data from 31 patients with LUSC were sourced from The Cancer Genome Atlas (TCGA) database.

### CT image preparation process

Following protocols established in prior research, CE-CT imaging was conducted using multi-slice spiral CT scanners [19]. Standard CT scans were performed before the administration of contrast media via syringe, followed by immediate CE-CT imaging. Raw CT images were retrieved from the picture archiving and communication system and then adjusted for optimal window settings. All images were annotated using Matlab 2018a (https://ww2.mathworks.cn/) and resized appropriately for the DL model input. Data augmentation techniques, including horizontal and vertical flips, 90° rotations, brightness and contrast adjustments, noise addition, color transformations, elastic deformations, and various mirror transformations, were applied exclusively to the training set to enrich the dataset. This approach was aimed at achieving a balanced and randomly distributed representation of images across categories without altering the validation cohorts, ensuring an authentic evaluation of model efficacy. Special attention was devoted to minimizing memory consumption during real-time augmentation and maintaining the original composition of the validation set to accurately reflect real-world performance. Moreover, additional augmentation methods such as random rotations, scaling, and cropping were considered, emphasizing the importance of optimizing both the model and augmentation parameters for improved training outcomes.

### MPR evaluation

Evaluation of the tumor's histological response involved quantifying the proportions of surviving tumor cells, necrotic tissue, and interstitial cells. MPR was characterized by less than 10% viable tumor cells remaining within the primary tumor bed, while the complete absence of viable tumor cells was indicative of a pathological complete response (CR) [20]. This assessment was conducted by two senior pathologists with over a decade of experience, independently reviewing all pathological samples. Any discrepancies were resolved through collaborative discussion to ensure an impartial analysis.

### ResNet50 model and implementation

**Figure 1.**
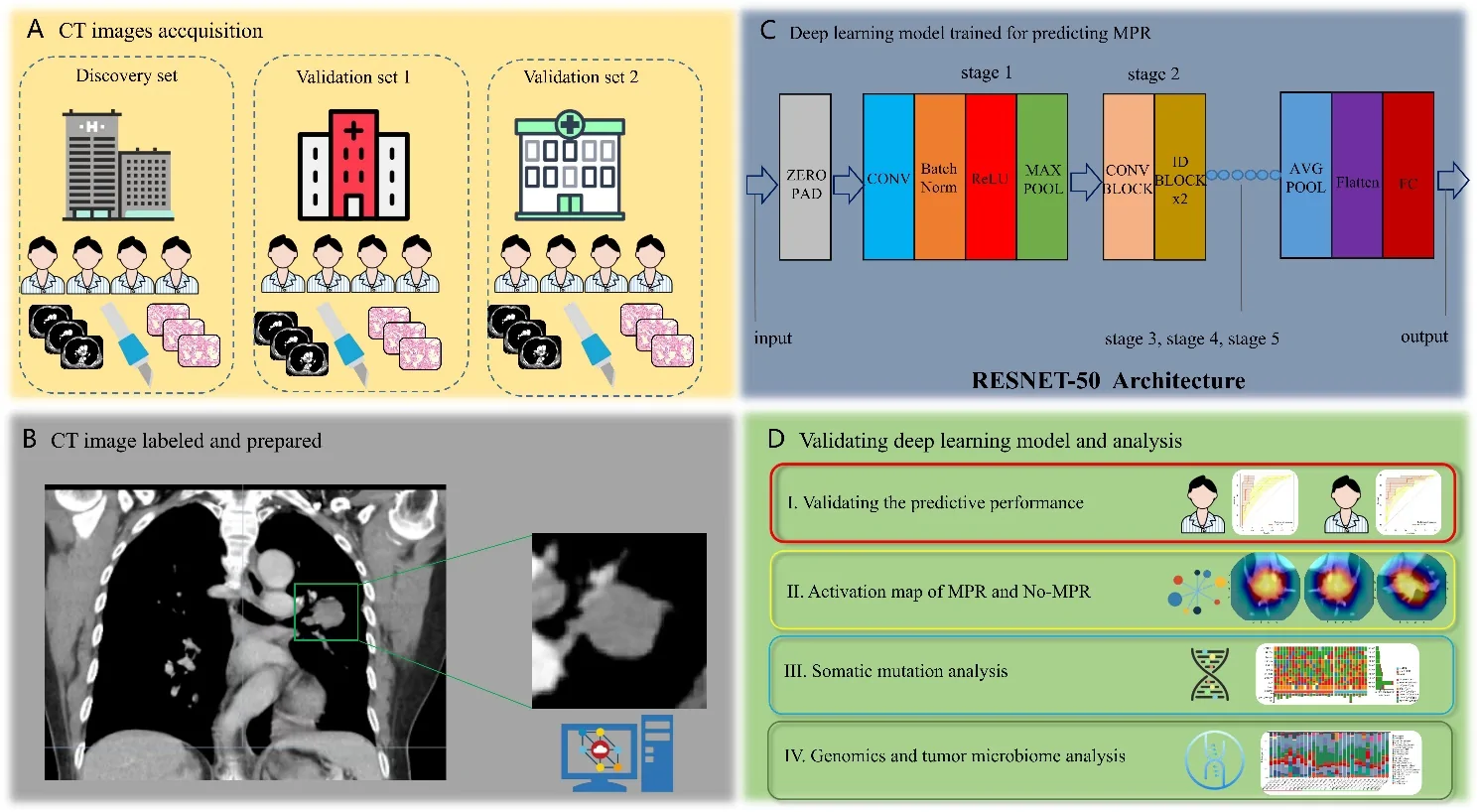
**Flowchart of the DL model to determine MPR and the mechanism analysis**. (**A**) The CE-CT image databases were collected from 309 patients with LUSC in three centers. (**B**) CE-CT images were labeled and prepared for DL model training. (**C**) Images were trained to predict MPR using the ResNet 50 model in the discovery set. (**D**) DL model was validated in the two validation sets, and the association between somatic mutations, gene expression, signaling pathways, immune cells, intra-tumoral microbes, and prediction models of NCI in patients with LUSC. DL, deep learning; MPR, major pathological response; CE-CT, contrast-enhanced computed tomography; LUSC, lung squamous cell carcinoma; NCI, neoadjuvant chemoimmunotherapy.

We constructed a deep convolutional neural network model aimed at accurately predicting the transformation from MPR to NCI (Fig. 1). To enhance the performance and stability of the model, we carefully selected ResNet50 as the underlying architecture. ResNet50, with its distinctive skip-connection technique integrating residual blocks, effectively promotes direct information flow, addressing the common issues of gradient vanishing and limited expressive capacity in deep networks. In ResNet50, each residual block consists of multiple convolutional layers, and these blocks are stacked together to form the entire network [21]. This stacking structure enables the model to progressively extract and learn from abstract representations of the data, better capturing complex features in the images. Specifically, each residual block contains a non-linear mapping, denoted as *F*(*x*, {*W_i_*}), representing the learning process from input feature map *x* to output feature map *y*. By introducing such mappings, the model can capture non-linear relationships in the data at different levels, enhancing its understanding of complex patterns. Ultimately, the output feature map *y* of the residual block can be precisely expressed through a mathematical formula, deepening our understanding of the internal operations of the model. The superiority of this structure lies in its ability to overcome challenges faced by many DL models when dealing with large-scale datasets, providing enhanced modeling capabilities for our prediction task. The output feature map of a residual block, *y*, can be mathematically expressed as follows:

$$y=F\left(x,\left\{W_{i}\right\}\right)+x$$

In this explanation, {*W_i_*} represents the weights of the residual block. The clever utilization of skip connections achieves direct propagation of residual information, where residual information refers to the difference between the input and output feature maps of the residual block. By adding the output of the residual block to the input, the network effectively captures residual information, thereby enhancing optimization during the learning process. This mechanism enables the model to learn and comprehend subtle differences in the input data, improving the network’s ability to represent complex features and injecting stronger learning capabilities for the ultimate predictive performance more precisely. The model’s architecture consists of convolutional and classification modules, achieved through three crucial steps: 1) establishing a seamless connection between a linear classifier and the convolutional network to create an efficient feature extractor; 2) freezing the convolutional network and connecting it to a fully connected network with 1,024 hidden nodes, enabling the integration of high-dimensional features for a high-precision classifier; and 3) unfreezing all network parameters to execute an end-to-end training approach with a lower learning rate.

In our study, ResNet50 was carefully chosen as the convolutional network, focusing on feature extraction from bounding box images. ResNet50 comprises five essential modules including Conv2D+Batch Norm+ReLu+Max Pool, Stage 2, Stage 3, Stage 4, and Conv5, collectively participating in the critical process of feature extraction. To enhance the integration of these extracted features, we introduced a fully connected network with an additional hidden layer containing 1,024 nodes. This hidden layer plays a crucial role in integrating features extracted from the convolutional network. Strategically employing the Softmax activation function, the output is transformed into a probability distribution, yielding the final two classification results. This strategic design further reinforces the model's performance, ensuring exceptional accuracy in the classification task. The provided description outlines the detailed structure of the ResNet-50, elucidating its components such as inputs, outputs, and the composition of layers in each stage. The architectural design of ResNet-50 aims to acquire more profound feature representations, significantly enhancing its performance across various tasks, including image classification and other computer vision applications. To ensure the correct implementation of the training program, we set the maximum epoch to 1000. This configuration not only facilitates the model in learning features more effectively but also provides enough training iterations to ensure the model's thorough convergence. It is noteworthy that we have employed PyTorch 2.0 as the implementation tool for the DL framework (https://pytorch.org/) and combined it with Python 3.8 (https://www.python.org/). Such a choice ensures code flexibility and readability while leveraging the powerful features of PyTorch, making the experimental design and implementation more efficient and convenient. The experimental setup was conducted in a Windows operating system environment, with system specifications comprising a 3.7 GHz Intel i7-12700KF CPU, NVIDIA GeForce RTX 3090, and 32 GB of RAM. This hardware configuration aims to provide sufficient computational power for the experiment, ensuring the model's efficient operation during both training and inference stages, while also guaranteeing the reproducibility and comparability of the experiment.

### Visualization technique of gradient-weighted Class Activation Mapping (Grad-CAM)

The core principle of Grad-CAM lies in quantifying the correlation between category predictions and feature maps from convolutional layers, ultimately generating class activation maps [22]. This technique accurately identifies crucial features and regions in the decision-making process of DL models, providing a powerful tool for model interpretability. Integrated with the previously described ResNet50 architecture, we further optimize the interpretability of the model. In the Grad-CAM framework, the initial steps involve executing forward propagation through a deep neural network (DNN) to generate predictions for the target category. This step, working in tandem with the ResNet50 model described earlier, collectively forms a more robust and comprehensive DL system. Subsequently, the back propagation algorithm extracts the gradients of the target category with respect to the intermediate feature maps, denoted by the symbol 'c', encompassing fundamental gradient information. The introduction of this step in Grad-CAM plays a crucial role, enabling a deeper understanding of the model's decision rationale for the target category. These gradient details complement the skip-connection technique in ResNet50, collectively building a comprehensive understanding of the internal operations of the model. Through this organic integration, we achieve more in-depth and accurate results in interpreting model decisions and visualizing essential features. This holistic approach not only enhances the interpretability capabilities of Grad-CAM but also further highlights the superiority of ResNet50 as the underlying architecture, providing more reliable and comprehensive interpretative support for the application of DL models. These gradients are denoted as c and represent essential gradient information:

$$\frac{\partial y_{c}}{\partial A^{k}}$$

where the variables *y_c_* and *A^k^* represent the predicted score of the target class c and the feature map of the k-th channel, respectively. The gradient values reflect the significance of different regions in the feature maps for the target class. Subsequently, we performed global average pooling on each channel of the feature maps to convert them into channel weights based on the following computation:

$$\alpha_{k}^{c}=\frac{1}{Z}\sum_{i}\sum_{j}\frac{\partial y_{c}}{\partial A_{ij}^{k}}$$

In this context, $a_{k}^{c}$ represents the channel weight and denotes a pixel in the feature map. The normalization constant Z is considered as well. Building upon the insights gained from evaluating the gradient weights in conjunction with the ResNet50 architecture, we derived the weights for individual channels, revealing the significance of different channels in predicting the target class. Higher weights assigned to specific channels indicate their substantial contributions to the prediction of the target class. To generate the definitive class activation map, we multiplied the gradient weights with their corresponding feature maps and conducted a channel-wise summation. This process, enriched by the interplay of Grad-CAM and ResNet50, leads to a comprehensive understanding of the model's decision-making process. The resulting map can be succinctly expressed as follows:

$$L_{Grad-CAM}^{c}=ReLU\left(\sum_{k}\alpha_{k}^{c}A^{k}\right)$$

Here, $L_{\mathrm{Grad}-\mathrm{CAM}}^{c}$ represents the class activation map for the target class 'c,' while ReLU denotes the activation function. The class activation map serves as an indicator of the network's attention distribution across different regions during the prediction of the target class. Brighter regions on the map correspond to more salient features, aligning with the earlier discussed principles. Following these principles, Grad-CAM effectively generates class-specific activation maps for the target class, offering detailed explanations and visual insights into the decision-making process of DNNs. By employing this methodology, we can achieve a more profound understanding of the decision rationale and identify key ROI within the network. As previously outlined, the integration of Grad-CAM with the ResNet50 architecture enhances the interpretability of the model and allows for a comprehensive exploration of significant features and decision-making factors in deep learning processes.

### Somatic mutation analysis

According to the classification of CT images, 31 patients with LUSC in this study were categorized into the following groups: P-MPR and P-No-MPR. Somatic mutation analysis was used to explore the potential association between genomics and P-MPR, and DNA sequence data (MAF format file) of patients with LUSC were downloaded from TCGA (www.cancer.gov/about-nci/GDC). The MAF file contains many fields ranging from chromosome names to cosmic comments. Additionally, in most somatic mutation analyses conducted using maftools, the selection primarily involved nine fields: Hugo_Symbol, Chromosome, Start_Position, End_Position, Reference_Allele, Tumor_ Seq_Allele2, Variant_ Classification, Variant_Type, and Tumor_Sample_Barcode. The top 10 genes with the highest mutational frequency were visualized using the “maftools” package. The TMB number and aneuploidy score were retrieved from cBioPortal (www.cbioportal.org/) for cancer genomics. Additionally, frequencies of mutational genes, TMB, and aneuploidy scores were compared between the P-MPR and P-No-MPR groups.

### Genomics analysis

Gene Set Enrichment Analysis was used to determine the association between the DL model and signal pathways and genomics expression [23]. The radiological data from 31 patients with LUSC were collected from The Cancer Imaging Archive (www.Cancer.Imaging.Archive.net/). Additionally, the CT images were classified into two groups based on DL model classification (P-MPR *vs* P-No-MPR) and used to screen differential gene expression (fold change ≥1.5, *P* < 0.05). Gene expression differential analysis was performed using the “edgR” package. Pathway analysis based on specific genes was used to understand the underlying mechanisms of the DL model (DAVID, https://david.ncifcrf.gov/). The analysis of Hub genes was then performed, and the top 10 genes were screened using Cytoscape software 3.6.1 (https://cytoscape.org/).

### Immune cell analysis in the TME

To assess the infiltration of immune cells in the TCGA database, the CIBERSORTx algorithm was employed, accessible at https://CIBERSORTx.stanford.edu/. This algorithm estimates the relative subset of RNA transcripts through genomics expression profiling and characterizes the cellular composition of complex tissues; its application enabled the assessment of immune cell infiltration in the TCGA database. Within the genomics data of LUSC, the Hugo symbols contained 20,531 gene probes, and the measurement of mRNA levels was conducted using RNAseq by expectation maximization in the TCGA dataset. To facilitate the analysis process, the gene expression file was appropriately processed for its transformation into a hybrid file format. Subsequently, a reference gene expression signature input matrix comprising 547 Hugo symbols, was created for estimating the relative proportion of 22 human immune cells. Finally, a phenotype class file was created, comprising a comparison of target classes between different samples. The prepared files were then uploaded to the CIBERSORTx online tool, and the results were acquired through the designated download link accessible at https://CIBERSORTx.stanford.edu/download.php.

### Lymphocyte sub-population analysis

The current investigation focused on the analysis of four distinct categories of circulating immune cells: natural killer T (NKT), B, natural killer (NK), and T cells. The identification of B cells relied on the expression of CD19, thus they are referred to as CD19^+^ B cells. Similarly, T cells were distinguished by their expression of CD3 were designated as CD3^+^ T cells. To further delineate subsets in the T lymphocytes, the presence of CD8 and CD4 markers was utilized. Furthermore, the T lymphocyte subsets were identified by the presence of CD8 and CD4 markers, denoted as CD3^+^ Cd8^+^ T and CD3^+^ CD4^+^ T cells, respectively. Additionally, T cells with a memory phenotype were distinguished through the presence of CD4 and CD45RO markers (referred to as CD4^+^ CD45RO ^+^ T cells), while CD4^+^ naïve T cells were characterized by the expression of CD4 and CD45RA (referred to as CD4^+^ CD45RA^+^ T cells). NKT and NK lymphocyte subsets were identified by assessing the combination of CD56 and CD3, with NKT cells defined as CD3^+^ CD56 ^+^ and NK cells as CD3^-^CD56^+^. Activated CD8^+^ T cells (known as CD8^+^ CD38^+^ T cells) were recognized by evaluating the presence of CD38 markers. The antibodies required for this experiment were supplied by BD Biosciences from San Jose, CA. These included CD56-FITC (#55664), CD4-FITC (#550628), CD8-FITC (#555366), CD45RA-PE (#555489), CD3-FITC (#555332), CD45RO-APC (#559865), CD19-FITC (#555412), and CD38-PE (#555460), as well as FITC/APC/PE controls (#55749; # 55748; and #55766). To perform lymphocyte staining, a 4 ml aliquot of peripheral blood was harvested from vessels to the tube containing acetic acid and anticoagulants. Afterward, five flow cytometry tubes were prepared with 20 μl of CD4-FITC CD45RO-APC CD45RA-PE, CD19-FITC, CD8-FITC CD38-PE, CD3-FITC CD56-PE, and FITC-PE APC isotype controls in separate tubes. Then, 100 ml of each blood sample was dispensed into the corresponding tube. Subsequently, the tubes were thoroughly mixed void of light to ensure proper antibody binding and incubated for a duration of 30 min at ambient temperature (20 °C). The hemolytic reagent was then (up to 2 ml; # 70-LSB3; BD Biosciences) was introduced to each tube. By conducting centrifugation (6 min), the supernatants were then eliminated. Next, two washes were performed using phosphate-buffered saline (# SH300256; Hyclone, Logan, UT), and the cells were resuspended in paraformaldehyde. Utilizing flow cytometry technology (FACSVIA; BD Biosciences), more than 2,000 cells in the lymphocyte gates were detected for each sample. The percentage of positive lymphocytes was examined using CellQuest PRO software (BD Bioscience).

### Intra-tumor microorganisms’ analysis

To explore the abundance of microorganisms in the LUSC, the details were downloaded from the Cancer Microbiome database (http://cancermicrobiome.ucsd.edu/CancerMicrobiome_DataBrowser/). Each patient had an average of 1,406 microorganisms including bacteria, archaea, and viruses. The differences in intra-tumor microbiome were compared between the two groups (P-MPR *vs* P-No-MPR). After identifying several pivotal cancer microbiomes, the correlation coefficients and heatmap were then respectively calculated and plotted using the “corrplot” package. Subsequently, the prediction model of P-MPR was constructed using logistic regression analysis based on the above selected cancer microbiomes.

**Table 1:** Characteristics of patients in the discovery and validation sets.

Variable	Patients N = 309 (%)	Discovery set (n = 200)	Validation set 1 (n = 60)	Validation set 2 (n = 49)	*P*-value^a^
**Age (years)**					0.661
**≤ 60**	93 (30.10%)	62 (31.00%)	19 (31.67%)	12 (24.49%)	
**> 60**	216 (69.90%)	138 (69.00%)	41 (68.33%)	37 (75.51%)	
**Gender**					0.811
**Female**	11 (3.56%)	6 (3.00%)	3 (5.00%)	2 (4.08%)	
**Male**	298 (96.44%)	194 (97.00%)	57 (95.00%)	47 (95.92%)	
**Smoking status**					0.910
**Smoker**	258 (83.50%)	166 (83.00%)	50 (83.33%)	42 (85.71%)	
**Non-smoker**	51 (16.50%)	34 (17.00%)	10 (16.67%)	7 (14.29%)	
**Stage**					0.743
**IIa**	16 (5.18%)	12 (6.00%)	3 (5.00%)	1 (2.04%)	
**IIb**	76 (24.60%)	50 (25.00%)	16 (26.67)	10 (20.41%)	
**IIIa**	217 (70.22%)	138 (69.00%)	41 (68.33%)	38 (77.55%)	
**Cycles**					0.708
**2**	222 (71.84%)	144 (72.00%)	41 (68.33%)	37 (75.51%)	
**3-4**	87 (28.16%)	56 (28.00%)	19 (31.67%)	12 (24.49%)	
**Radiological response**					0.867
**CR**	71 (22.97%)	48 (24.00%)	15 (25.00%)	8 (16.32%)	
**PR**	153 (49.51%)	96 (48.00%)	31 (51.67%)	26 (53.06%)	
**SD**	74 (23.95%)	48 (24.00%)	12 (20.00%)	14 (28.57%)	
**PD**	11 (3.57%)	8 (4.00%)	2 (3.33%)	1 (2.05%)	
**Pathologic response**					0.550
**MPR**	149 (48.22%)	96 (48.00%)	32 (53.33%)	21 (42.85%)	
**No-MPR**	160 (51.78%)	104 (52.00%)	28 (46.97%)	28 (57.15%)	

a *P*-value is derived from the difference in clinical characteristics between the discovery data set and the two validation data sets. CR, complete response; PR, partial response; SD, stable disease; PD, progressive disease; MPR, major pathological response.

### Statistical analysis

The distribution of frequencies was determined based on the baseline characteristics of subjects with LUSC who underwent NCI, and a Chi-square test was employed to compare these frequencies. To evaluate the predictive capability of the DL model, the area under receiver operating characteristic curve (AUC) was utilized as a primary metric. The AUC was calculated using the “pROC” package, while the ROC curve was plotted using the “ggplot2” package. Additionally, the model performance was evaluated through the Akaike Information Criterion (AIC) scores, in which a lower score reflected a superior predictive ability. The analysis of MPR involved the utilization of the "rms" package for multivariate analysis. To compare the frequencies of somatic mutations, the Chi-square test was employed. Predictive score, TMB, aneuploidy score, and immune cell percentage were assessed using the Mann-Whitney U test. To select the cancer microbiomes with optimal non-zero coefficients, we employed the least absolute shrinkage and selection operator (LASSO) algorithm with a five-fold cross-validation approach. All statistical analyses were performed using R (version 3.5.2; www.R-project.org) and GraphPad Prism 9 (www.graphpad.com/). The criteria for statistical significance were set at a level of *P* < 0.05.

## RESULTS

### Clinical characteristics of patients

All patients (n=309) underwent R0 curative surgery. The basic clinical features of individuals with LUSC treated with NCI in the discovery set, validation set 1, and validation set 2 are presented in Table 1, with 138 (69.00%), 41 (68.33%), and 37 (75.51%) patients aged >60 years, respectively. Among the three cohorts, 194 (97.00%), 57 (95.00%), and 47 (95.92%) patients were male, respectively. Most individuals in the discovery set (83.00%), validation set 1 (83.33%), and validation set 2 (85.71%) cohorts were current or previous smokers. Our results revealed that the three cohorts shared similar clinical stages (IIa: 6.00% *vs* 5.00% *vs* 2.04%; IIb: 25.00% *vs* 26.67% *vs* 20.41%; and IIIa; 69.00% *vs* 68.33% *vs* 77.55%, respectively). In the discovery set, validation set 1, and validation set 2, 144 (72.00%), 41 (68.33%), and 37 (75.51%) individuals mainly had two cycles, respectively. Among the three cohorts, 48 (24.00%), 15 (25.00%), and 8 (16.32%) patients achieved complete response (CR), while 96 (48.00%), 31 (51.67%), and 26 (53.06%) achieved partial response (PR), respectively. Additionally, 96 (48.00%), 32 (53.33%), and 21 (42.85%) achieved MPR to neoadjuvant chemo-immunotherapy.

### DL model development and validation in patients with LUSC undergoing NCI before surgery

**Figure 2.**
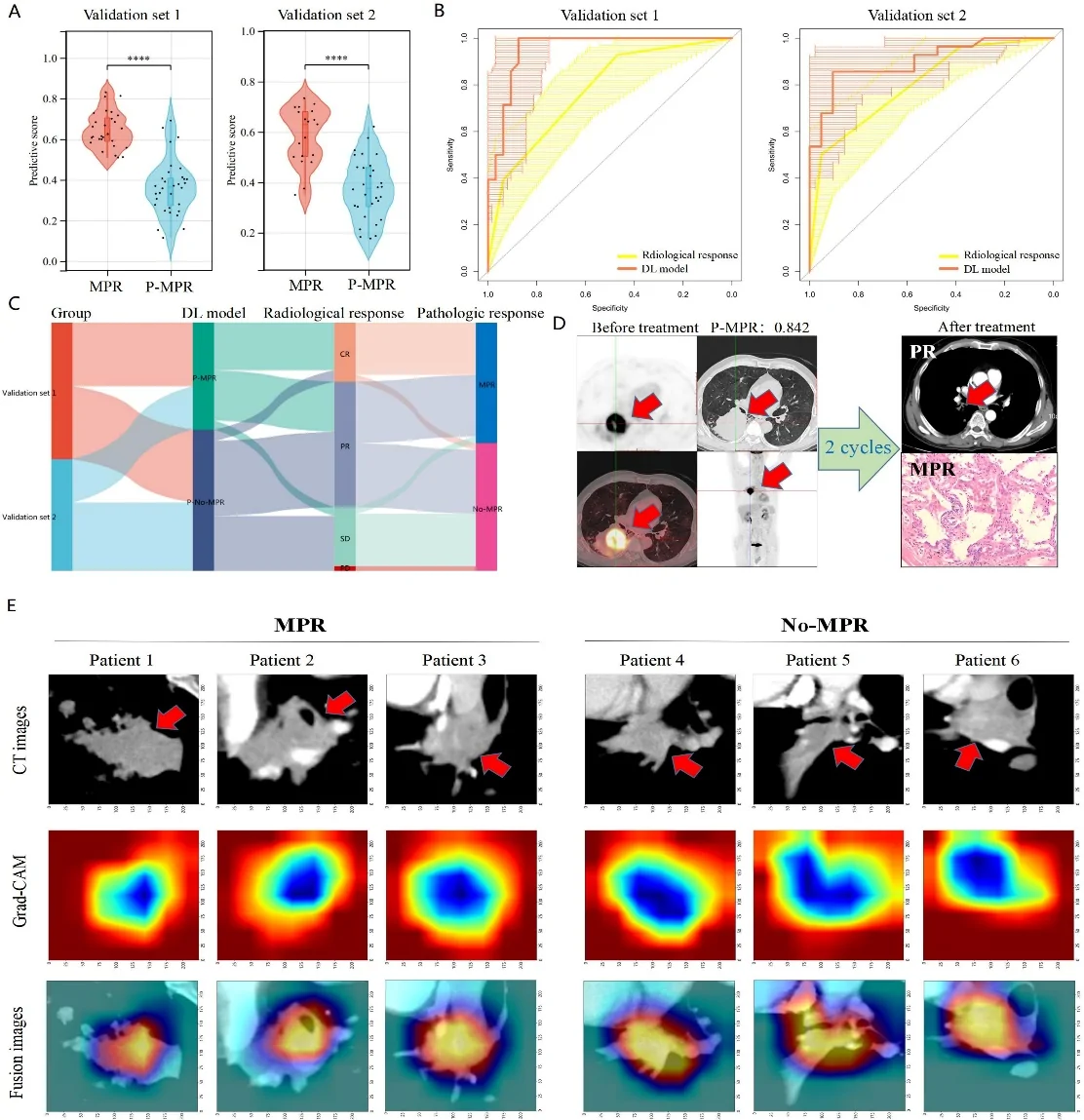
**Predictive performance of the DL model and activation mapping**. (**A**) Predictive score was compared between the MPR and No-MPR in the two validation sets. (**B**) Predictive performance of the DL model and radiological response for estimating MPR were shown as ROC curves. (**C**) Correlation analysis of P-MPR, radiological response, and MPR are presented as a Sankey diagram. (**D**) An example of the patient with MPR and PR receiving NCI. (**E**) Row 1 shows the input image of the DL model from MPR and No-MPR patients. The darker blue areas of Grad-CAM images with the most contribution to maximizing the outputs of the final prediction layer in row 2. The yellow areas of fusion images represent the important predictive area of CT images in row 3. DL, deep learning; MPR, major pathological response; P-MPR, predictive MPR; ROC, receiver operating characteristic curve; CT, computed tomography; Grad-CAM, Gradient-weighted Class Activation Mapping.

The DL model based on ResNet50 was trained in the discovery set, and the training process was shown as accuracy (Supplementary Fig. 1A) and cross-entropy loss curves (Supplementary Fig. 1B). The model demonstrated a strong performance with an AUC and accuracy of 0.953 (Supplementary Fig. 1C) and 93.00% (Supplementary Fig. 1D), respectively. Patients evaluated as MPR were associated with a significantly higher predictive score than those with No-MPR in validation sets 1 and 2 (both *P* < 0.001) (Fig. 2A). Subsequently, the DL model was tested, and the two cohorts presented high AUC values of 0.95 (95% confidence interval [CI]: 0.98-1.00) and 0.90 (95% CI: 0.81-0.98). Subsequently, the final cutoff value of the DL score was calculated as 0.48 based on the Youden Index. The sensitivities of validation sets 1 and 2 were 87.50% (95% CI: 71.01%-96.49%) and 85.71% (95% CI: 67.33%-95.97%), respectively. The specificity of the two cohorts was 100.00% (95% CI: 87.66%-100%) and 90.48% (95% CI: 69.62%-98.83%), respectively. We found that the radiological response for predicting MPR showed lower AUC values [0.77 (95% CI: 0.65-0.89) and 0.80 (95% CI: 0.68-0.92), both *P* < 0.001] than that for the DL model in the validation sets 1 and 2 (Fig. 2B). The AIC scores were 134.09 and 86.144 in the radiological response and DL model, respectively. The lower AIC score of different models indicated better predictive performance. We further explored the association between the DL model, radiological response, and pathological response. Patients with P-MPR (n = 47) mainly demonstrated CR (42.55%) or PR (48.93%) and MPR (91.48%) status in the combination of validation sets 1 and 2 (Fig. 2C). A case showed that the patient had a high predictive score (P-MPR: 0.842) and acquired MPR validated through radical operation after two cycles of NCI (Fig. 2D).

**Figure 3.**
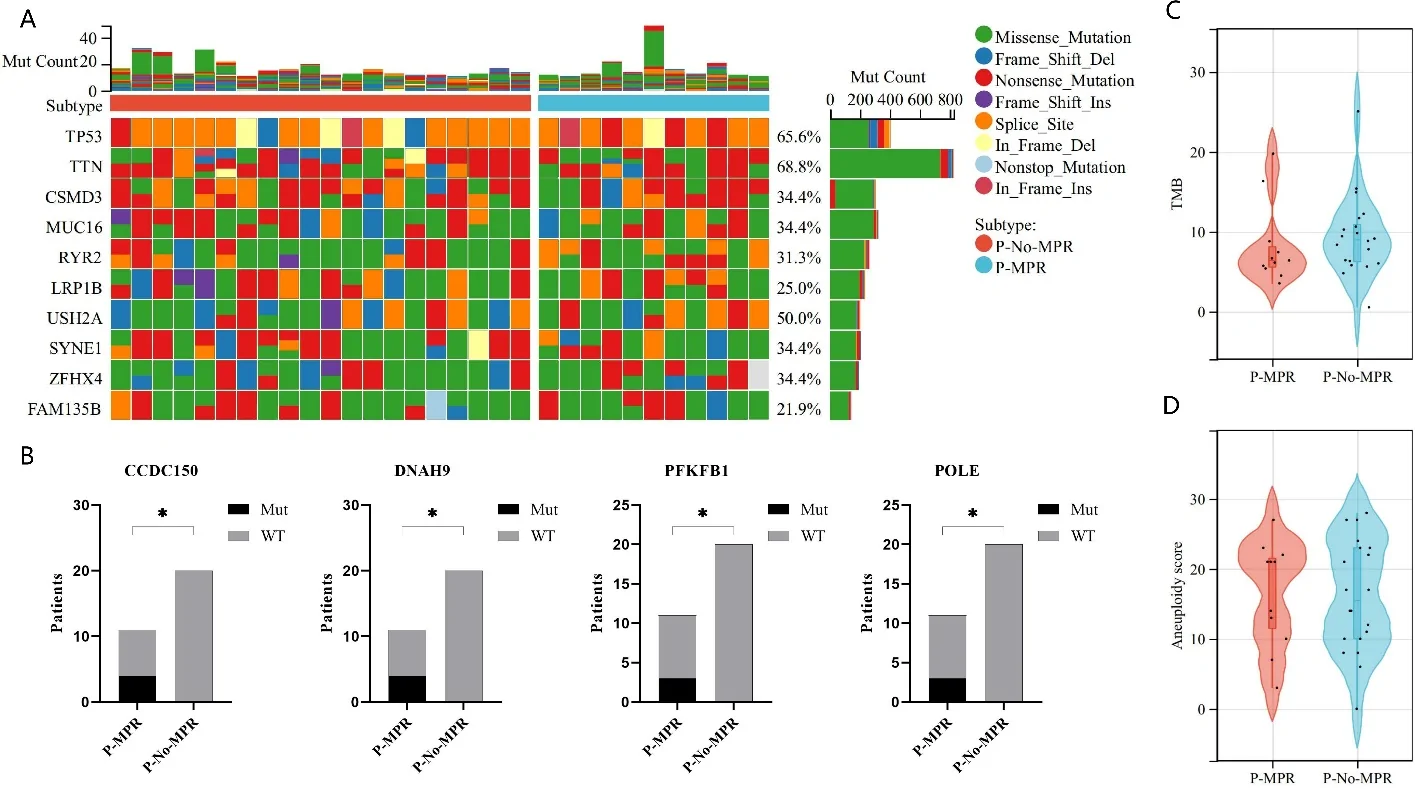
**Association between the DL model and somatic mutations**. (**A**) Summary of molecular features associated with P-MPR in TCGA-LUSC set. (**B**) Frequencies of somatic mutations were compared between P-MPR and P-No-MPR groups. (**C**) TMB was compared between P-MPR and P-No-MPR groups. (**D**) Aneuploidy score was compared between P-MPR and P-No-MPR groups. DL, deep learning; P-MPR, predictive MPR; P-No-MPR, predictive No-MPR; TCGA, The Cancer Genome Atlas; LUSC, lung squamous cell carcinoma; TMB, tumor mutation burden.

Multivariate logistic regression analysis revealed that the radiological response (CR/PR *vs* stable disease [SD]/progressive disease [PD]) and DL model (low score *vs* high score) were independent predictive factors [OR: 6.17 (95% CI: 1.46-33.65) and 46.06 (95% CI: 14.27-191.17), *P* = 0.02 and <0.001, respectively]. Moreover, we evaluated the performance stability of the DL model in the different subgroups of clinical characteristics (Supplementary Table 1). The DL model achieved relatively robust and satisfactory predictive ability in most subgroups such as age (≤ 60 years: AUC = 0.97; >60 years: AUC = 0.91), smoking status (smoker: AUC = 0.93; non-smoker: AUC = 0.93), TNM stage (stage II: AUC = 0.85; stage III: AUC = 0.96), cycles (2: AUC = 0.95; 3-4: AUC = 0.86), and radiological response (CR/PR: AUC = 0.91; SD/PD: AUC = 0.92). The remaining subgroups had weak aspects of the DL model; for example, the female subgroup (AUC = 1.00, *P* = 0.157). The primary reason was the relatively small sample size of total female patients with LUSC (n = 8).

### MPR and No-MPR CT image visualization using Grad-CAM class activation

The activation map of the DL model—specifically to the final convolution layer—was generated for both MPR and No-MPR patients to comprehend the regions influencing the DL model predictions in the CT images (Fig. 2E). The gradient size within this layer served as a metric for the "importance" of each node or voxel concerning the final prediction layer. Building upon the insights gained from the ResNet50 architecture and Grad-CAM framework, this analysis provided a nuanced understanding of the critical regions influencing predictions, encompassing both within and outside the tumor. In both Grad-CAM images, the darker blue hues indicated a higher predictive score. Additionally, fusion images were employed to investigate the focal areas of interest for the DL model. In these images, yellow highlights represented the significant regions influencing the DL model's prediction of MPR or No-MPR.

### Somatic mutations associated with MPR prediction of DL model

The same DL algorithm in the discovery set was used in CT images of the 31 patients with LUSC, and all individuals were categorized into P-MPR (N = 11) and P-No-MPR (N = 20) groups according to the above cutoff values (0.48). To explore the association between somatic mutations and P-MPR using DNA sequence data, the frequency of mutation genes <1% was removed, and the top 10 genes of high mutational frequency included TTN (68.80%), TP53 (65.60%), CSMD3 (34.40%), and MUC16 (34.40%). Among single nucleotide variants there were eight mutational types such as missense mutation frameshift del, nonsense mutation, frameshift ins, splice site, in frame del, nonstop mutation, and in frame ins (Fig. 3A). Overall, 1,601 genes were analyzed using the LASSO method. Ultimately, four mutational genes were found to be positively related to P-MPR, namely, CCDC150 (36.36% *vs* 00.00%, χ^2^ = 8.35, *P* = 0.003), DNAH9 (36.36% *vs* 00.00%, χ^2^ = 8.35, *P* = 0.003), PFKFB1 (27.27% *vs* 00.00%, χ^2^ = 6.03, *P* = 0.014), and POLE (27.27% *vs* 00.00%, χ^2^ = 6.03, *P* = 0.014) (Fig. 3B). TMB and aneuploidy scores were also compared between P-MPR and P-No-MPR groups, and we found no association (*P* = 0.529 and 0.881, respectively) (Fig. 3C and D).

**Figure 4.**
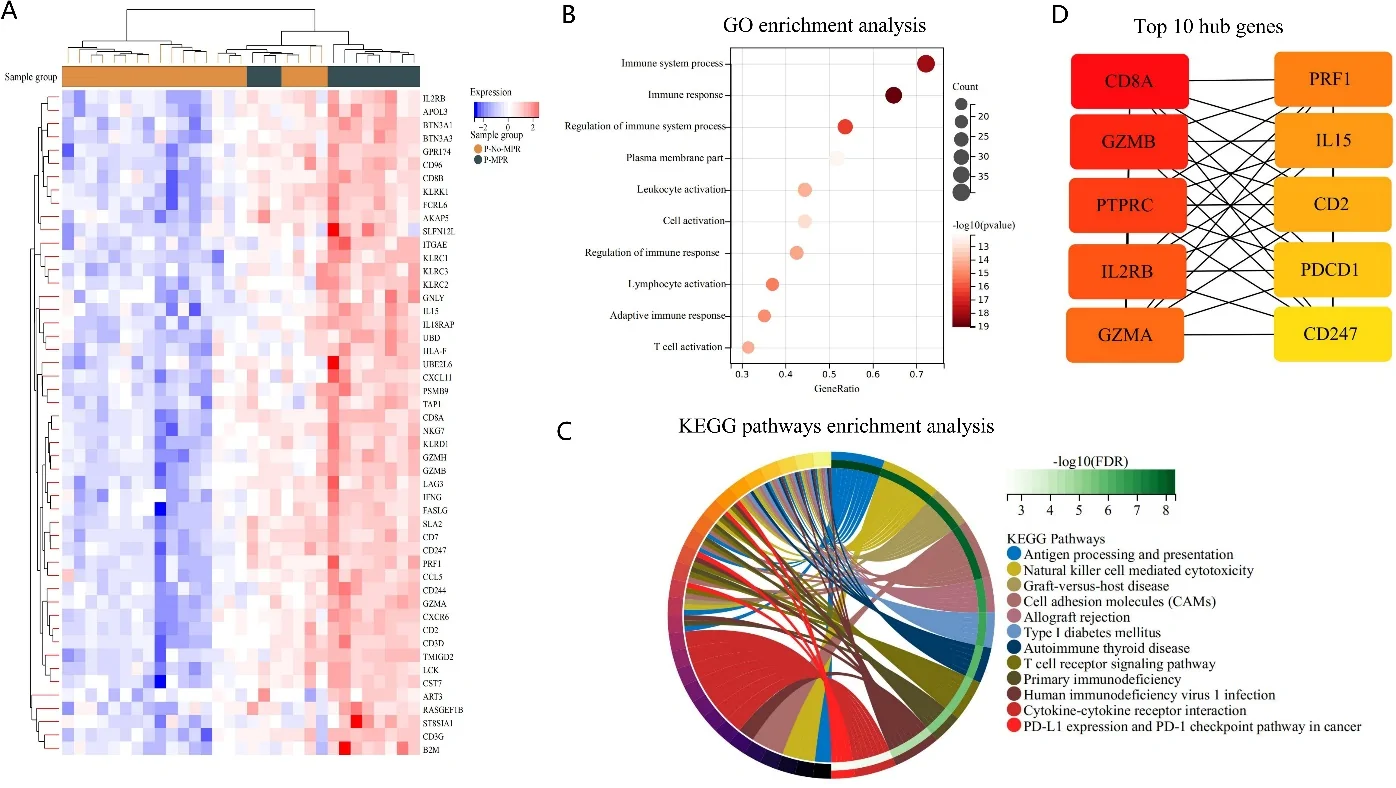
**Genomics analysis for investigating the potential biological basis of the DL model**. (**A**) Heatmap presenting the differentially expressed genes in samples compared to P-MPR with P-No-MPR groups. (**B**) A bubble plot of the top 10 enriched Gene Ontologies (GO). (**C**) KEGG pathways identified using gene enrichment analysis for the set of differentially expressed genes. (**D**) Hub genes screened using Cytoscape. DL, deep learning; P-MPR, predictive MPR; P-No-MPR, predictive No-MPR; KEGG, Kyoto Encyclopedia of Genes and Genomes.

### Genomics expression difference and biological basis analysis

Based on the differential gene expression analysis (P-MPR *vs* P-No-MPR), 1,581 genes were significantly up-regulated (*P* < 0.05) (Fig. 4A) including PRF1, CD8A, GZMH, GZMA, GZMB, and CD8B (Supplementary Fig. 2). Additionally, 14 genes were up-regulated after FDR-adjusted *P*-value including the BTN3A1 (*P* = 0.003), CD8A (*P* = 0.011), SLA2 (*P* = 0.011), CD3G (*P* = 0.011), and CXCR6 (*P* = 0.011) (Supplementary Table 2). Gene Ontology (GO) enrichment analysis revealed that those genes were associated with the immune system process, immune response, and regulation of the immune system process (Fig. 4B). Several Kyoto Encyclopedia of Genes and Genomes (KEGG) pathways were significantly associated with P-MPR, such as antigen processing and presentation, NK cell-mediated cytotoxicity, graft versus host disease, and cell adhesion molecules (Fig. 4C). In the analysis of hub genes, the top 10 genes were screened and considered to potentially play important roles in the MPR occurrence of patients with LUSC with NCI (Fig. 4D). Moreover, no difference was found in the OS and disease-free survival between P-MPR (n=11) and P-No-MPR (n=20) groups in patients with LUSC receiving radical operation without immunotherapy (hazard ratio [HR] = 0.433 [0.107-1.754], *P* = 0.286; and HR = 0.374 [0.052-2.664], *P* = 0.375, respectively) (Supplemntary Fig. 3).

### Immune cells and lymphocyte sub-population correlated with MPR prediction

**Figure 5.**
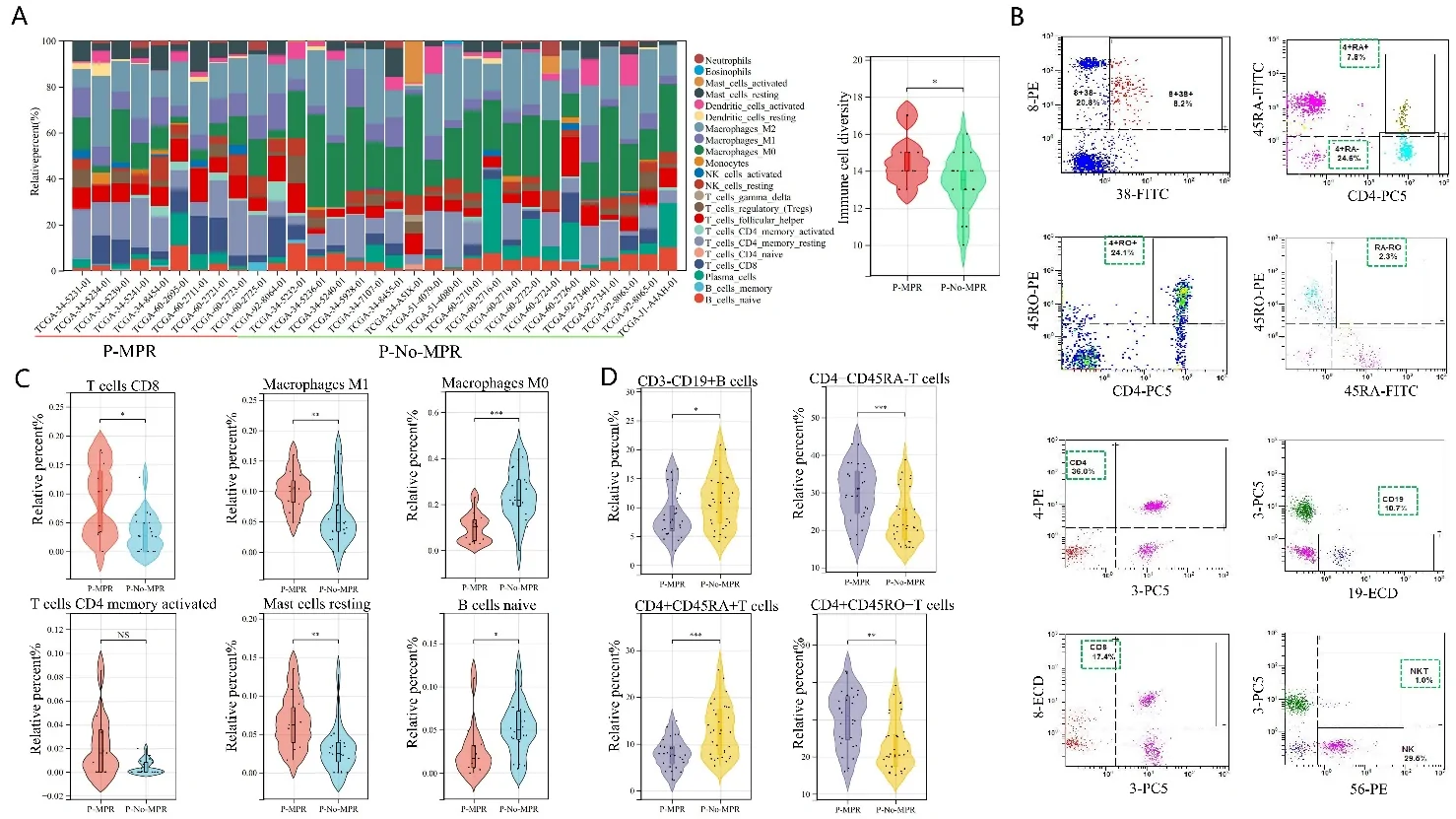
**Correlation between immune cells and P-MPR**. (**A**) Percentage and diversity of immune cells via CIBERSORTx analysis are shown in the P-MPR and P-No-MPR groups. (**B**) Flow cytometric cell population profiling was presented. (**C**) Intra-tumor immune cells were compared between P-MPR and P-No-MPR groups. (**D**) Immune cells of peripheral blood were compared between P-MPR and P-No-MPR groups. P-MPR, predictive MPR; P-No-MPR, predictive No-MPR.

To explore the association between immune cells and P-MPR, we analyzed the composition and abundance of 22 types of immune cells and their diversity in the TME (Fig. 5A) including T cell CD8^+^, neutrophils, eosinophils, and NK cells activated. We found that the P-MPR group showed more categories of immune cells than the P-No-MPR group in the patients with LUSC (*P* = 0.025). Significant negative or positive associations existed among all subtypes of immune cells (Supplemntary Fig. 4). Naïve T cells were associated with activated mast cells (r= 0.92, *P* < 0.001) and T cell gamma delta (r= 0.96, *P* < 0.001). A negative association was found between T cell CD8^+^ and macrophage M0 (r = -0.66, *P* < 0.001). The percentage of peripheral blood lymphocyte subtypes was determined using flow cytometric analysis, and different flow cytometric cell population profiling was presented (Fig. 5B). The percentages of T cell CD8, macrophage M1, and mast cells resting were significantly higher in P-MPR patients than those of the above immune cells in P-No-MPR patients (*P* = 0.010, < 0.001 and < 0.001, respectively) (Fig. 5C). In contrast, the percentages of macrophage naïve and naïve B cells were significantly lower in P-MPR patients than those of the above immune cells in P-No-MPR patients (*P* < 0.001 and *P* = 0.010, respectively). The percentage of T cell CD4 memory activated was not different between P-MPR and P-No-MPR groups (*P* = 0.07). We also found that the percentages of CD3-CD19^+^ B and CD4^+^ CD45RA^+^ T cells were significantly higher in P-MPR patients than those of the above circulating immune cells in P-No-MPR patients (*P* = 0.040 and < 0.001, respectively) (Fig. 5D). The frequencies of CD4^+^ CD45RA^-^ T and CD4^+^ CD45RO^+^ T cells were significantly lower in P-MPR patients than those of the above circulating immune cells in P-No-MPR patients (*P* < 0.001 and *P* = 0.001, respectively).

### Intra-tumor microorganisms’ analysis

**Figure 6.**
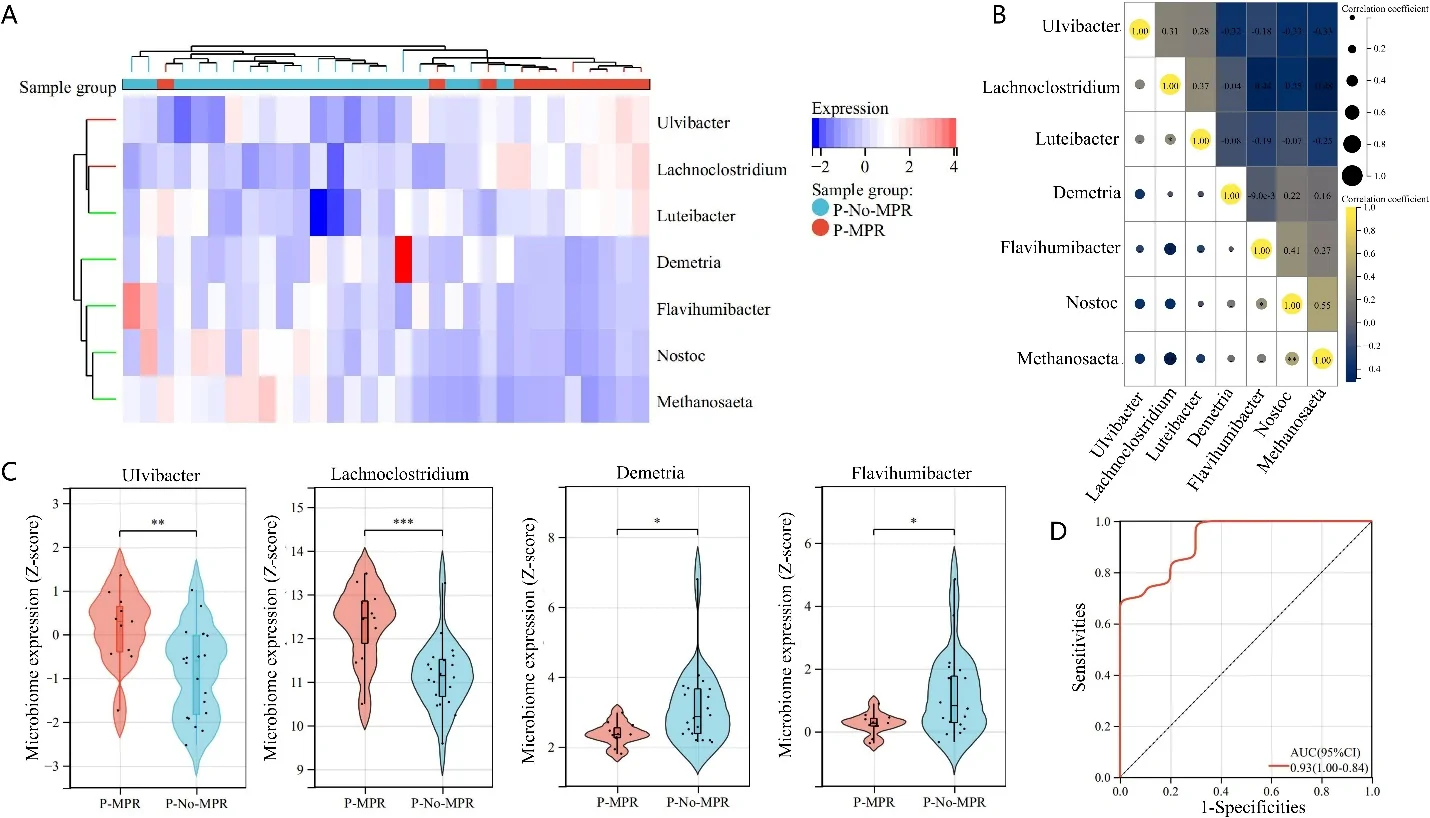
**Correlation between intra-tumor microorganisms and P-MPR**. (**A**) Heatmap presents the differential abundances in samples compared to P-MPR with P-No-MPR groups. (**B**) Association of each cancer microbiome was shown. (**C**) Abundances of intra-tumor microbiomes were compared between the P-MPR and P-No-MPR groups. (**D**) P-MPR model was constructed using four intra-tumor microbiomes and plotted using the ROC curve. P-MPR, predictive MPR; P-No-MPR, predictive No-MPR.

To understand the underlying relationship between the cancer microbiomes and P-MPR in the TME, we analyzed 1,000 microorganisms and found seven species with significant differences such as *Ulvibacter*, *Demetria*, and *Methanosaeta* (Fig. 6A). The correlations among seven different cancer microbiomes are shown in Fig. 6B. Associations were found between *Luteibacter* and *Lachnoclostridium* (r=0.31, *P* =0.090), *Methanosaeta* and *Nostoc* (r=0.68, *P*<0.001), *Nostoc* and *Flavihumibacter* (r= 0.58, *P* < 0.001), *Methanosaeta* and *Flavihumibacter* (r=0.47, *P*=0.008), and *Flavihumibacter* and *Lachnoclostridium* (r=0.50, *P*=0.004) (Supplemntary Fig. 5). The LASSO method was used to further screen the optimal four species. The microbiome expression percentages of *Ulvibacter* and *Lachnoclostridium* were significantly higher in P-MPR patients than those of the above two microbiome expressions in P-No-MPR patients (*P* = 0.004 and < 0.001, respectively) (Fig. 6C). The logistic model constructed to explore the MPR prediction using cancer microbiomes used the formula in Supplementary Table 3 and presented a high AUC value of 0.93 (95% CI: 0.85-1.00) (Fig. 6D). The cutoff value of the predictive score was calculated as 0.44 based on the Youden Index. The sensitivity specificity was 85.00% (95% CI: 62.11%-96.79%) and 81.82% (95% CI: 48.22%-97.72%), respectively.

**Table 2:** Univariate and multivariate analyses for MPR in the discovery set.

Variable	Univariate analysis		Multivariate analysis	
	OR (95% CI)	*P*-value	OR (95% CI)	*P*-value
**Age (years) (≤ 60 *vs.* > 60)**	0.471 (0.194-1.098)	0.086	--	--
**Gender (female *vs.* male)**	4.489 (0.637-9.432)	0.186	--	--
**Smoking status (smoker *vs.* non-smoker)**	1.227 (0.432-3.538)	0.699	--	--
**Stage (II *vs.* III)**	1.297 (0.558-3.042)	0.545	--	--
**Cycles (2 *vs.* 3-4)**	1.457 (0.632-3.429)	0.380	--	--
**Radiological response (CR/PR *vs.* SD/PD)**	9.87 (3.440-35.995)	< 0.001*	6.174 (1.463-33.651)	0.020*
**DL model (Low score *vs.* High score)**	55.900 (18.088-219.777)	< 0.001*	46.063 (14.273-191.172)	< 0.001*

OR, odds ratio; CI, confidence interval; CR, complete response; PR, partial response; SD, stable disease; PD, progressive disease; DL model, deep learning model; AIC, Akaike Information Criterion. *P-value < 0.05.

## DISCUSSION

Compared to traditional neoadjuvant chemotherapy, NCI exhibits significant potential for enhancing the likelihood of radical resection and improving prognosis for NSCLC. However, a substantial number of NSCLC patients do not achieve an MPR with neoadjuvant chemotherapy despite considerable advancements. In this study, we harnessed the power of DL methods utilizing multicenter CT images to develop predictive models for MPR in LUSC patients undergoing NCI. The DL model demonstrated a notable AUC value for predicting P-MPR in two independent validation sets. Visualization was enhanced through the fusion of CT and Grad-CAM images, presenting an insightful display of the DL model's attention to important areas. Somatic mutations, genomics, immune cell profiles, and intra-tumor microorganisms were subjected to analysis, and the underlying mechanisms of the DL model were elucidated through comprehensive multi-omics research.

Finding biomarkers for NCI in lung cancer has been an increasingly hot area of research. Recently, the pretreatment radiomics model has been built, with the AUC value of 0.82 (95% CI: 0.72-0.93) of P-MPR in the validation [24]. Additionally, the radiomics-based nomogram for predicting MPR to NCI in patients with potentially resectable NSCLC was improved to have the AUC value of 0.84 (95% CI: 0.74-0.93). This radiomics model proposed that the CT images could also be used to predict MPR and the AUC value in our DL model was higher than that in both validation sets 1 and 2 in this radiomics model. The pilot study reported that multiparametric magnetic resonance imaging could be used to evaluate pathological response to the NCI in resectable NSCLC and that combining intravoxel incoherent motion diffusion-weighted imaging and diffusion kurtosis imaging quantitative parameters achieved the best performance AUC value of 0.889 [25]; however, the samples in this study were relatively small and lacked validation data. Furthermore, the efficiency of 18F-fluorodeoxyglucose (18F-FDG) positron emission tomography-CT for predicting the MPR to the neoadjuvant PD-1 blockade was explored in resectable NSCLC, and it was found that 18F-FDG uptake could predict MPR in patients with NSCLC with neoadjuvant immunotherapy [26, 27]. However, those models did not use the DL method to build the model, and extensive radiological information has not been mined. In our study, we employed DL to predict the treatment response using CT images and found that the DL model had a better predictive value of MPR in patients with LUSC. These studies demonstrated that the DL based on pretreatment CT images had important predictive value in NCI and was superior to conventional machine learning of radiomics or image parameters. As DL has a black box effect [10], interpreting the model becomes challenging, especially when applied in the field of medicine. Therefore, we employed the Grad-CAM method to understand the important area of P-MPR using CT image visualization, and the DL model primarily focused on the tumor area rather than the normal lung tissue. These findings revealed that the DL model predicted the MPR or No-MPR in patients with LUSC by focusing on the core areas of the tumor, and using most of the tumor CT images based on the DL features may be a promising method for treatment response prediction.

The DNA sequence database has been increasingly and extensively used in cancer research, and most studies have found a close association between genomics variants and prognosis or treatment outcomes [28-35]. For example, the somatic mutations of *EGFR* and *STK11* had poorer immunotherapy outcomes than *EGFR* and *STK11* wide-type. In contrast, *TP53* and *KRAS* had better immunotherapy outcomes than TP53 and KRAS wide-type [14]. In this study, we used the DL model to explore the relationship between somatic mutations and MPR prediction. Notably, CCDC150, DNAH9, PFKFB1, and POLE mutations were significantly associated with P-MPR; however, TMB and aneuploidy scores were not associated with P-MPR. According to previous studies, POLE mutation was significantly associated with immunotherapy in pan-cancers [36, 37]; however, further validation with a larger patient cohort is required to establish this relationship with certainty at the NCI. We further analyzed the different genes between P-MPR and P-No-MPR groups. Inflammation-related genes such as IFNG, GZMB, and NKG7 related to the cytotoxic activity of T and NK cells were also found in the TME of pathological response in resectable patients with NSCLC treated with NCI, consistent with previous studies [38, 39]. The immune system process and immune response were significantly up-regulated in the P-MPR using GO analysis. Additionally, differential pathways including antigen processing and presentation, were up-regulated in MPR tumors using KEGG analysis, consistent with improved CPR in patients with NSCLC undergoing NCI in other studies [39]. Hub genes were further analyzed, and active immune biomarkers of CD8A, GZMB, and GZMA were identified. These findings suggest that the inflammation status of the pretreatment tumor immune microenvironment could lead to the release of more pro-inflammatory factors than the no-inflammation status, which would significantly improve the pathological response in patients with LUSC undergoing NCI.

Immune cells are closely related to the outcome of tumor therapy, and changes in the abundance of immune cells have been revealed in pretreatment and posttreatment tumor samples [40]. After patients with stage IIIA NSCLC are treated with NCI, increasing B cells and CD4+ T cells based on single-cell RNA-sequencing analysis from radical surgery samples have been found to be associated with a positive therapeutic response [41]. Interestingly, in this study we found elevated levels of T cell CD8 and macrophage M1 in the P-MPR pretreatment samples, while elevated levels of naïve B cells were observed in the P-No-MPR pretreatment samples. The T cell CD8 plays an important role in the killing of tumors, and the abundance has been correlated with the efficacy of immunotherapy [42]. Additionally, macrophage polarization is a different functional state of macrophages in different environments, and macrophage M1 has strong pro-inflammatory and antigen-presenting abilities [43]. We speculate that the T cell CD8 and macrophage M1 activities in the posttreatment TME further led to the release of inflammatory cytokines and the recruitment of different immune cells to kill tumor cells. The differential fractions of these immune cells in pretreatment tumors may be one biomarker to predict the therapeutic efficacy of NCI. Besides tumor immune cells, peripheral blood immune status was also crucial and could be used to evaluate the clinical benefits of anti-PD-1/PD-L1 therapy [44, 45]; for example, specific PD-1^+^ CD56^+^ and CD8^+^ T cells frequently indicate a favorable prognosis in patients with melanoma treated with immunotherapy [46]. Similarly, in our study, CD4^+^ CD45 RA^-^ T and CD4^+^ CD45RO^+^ T cells were significantly high in the patients with LUSC of P-MPR, suggesting that the circulation of CD4^+^ T cells plays an important role in enhancing the response to NCI. In addition to immune cells, the potential of the microbiota within a tumor to be critical for tumor initiation and progression has not been reported regarding neoadjuvant therapy [47]. Therefore, we used DL to mine the biological information behind the images in our study and found that several tumor microorganisms were closely related to P-MPR, particularly for *Lachnoclostridium*. The association between *Lachnoclostridium* and objective responses of immunotherapy in hepatocellular carcinoma, as well as the diagnostic biomarker based on the low abundance of *Lachnoclostridium* in colorectal cancer, have been previously reported [48, 49]; however, the association of intra-tumoral *Lachnoclostridium* with NCI for lung cancer was first reported. These findings suggest that high *Lachnoclostridium* levels contribute to the occurrence of therapeutic responses in patients with LUSC undergoing NCI; however, the relationship between the other three microbiomes and lung tumors is poorly understood and needs further investigation. Moreover, we established the prediction model of P-MPR based on four kinds of microorganisms in tumors and achieved good accuracy; however, the model performance of predicting MPR needs further validation using external data.

This study had some limitations. First, the study primarily focused on patients with LUSC and the number of samples was relatively small. Because squamous cell carcinoma and adenocarcinoma differ in both genomics and CT imaging information, the performance may not be ideal for MPR prediction in adenocarcinoma. Although we used two validation cohorts to test the predictive accuracy of the DL model, more patients should be recruited and validated. Second, the follow-up time of our patients was short, and the patient’s prognostic information was unavailable, leading to the unclear relationship between MPR predicted using the model and PFS or OS. Therefore, studies with longer follow-ups are needed to further confirm the clinical value of this model. The publication of more clinical trial results may also help to discover the relationship between pathological remission and prognosis. Third, the "black boxes" related to artificial intelligence are challenging for directing future research. The Grad-CAM method cannot fully explain the DL model, but the Grad-CAM is still an excellent algorithm for most image analysis fields. Fourth, we used a single CT image dataset, and sequencing data were mainly used to perform biological interpretation of the DL model. The DL model did not include information on pretreatment pathology images, RNA, or DNA sequencing data, which resulted in some deficiencies in multi-omics prediction.

In this study we presented a novel prediction model of neoadjuvant therapy for LUSC with good prediction accuracy, in which the DL model was shown via fusion CT imaging to focus primarily on the tumor area to assess treatment response. In contrast to previous models, this study marks the first instance in which we analyzed the association between somatic mutations, gene expression, signaling pathways, immune cells, intra-tumor microbes, and prediction models of NCI in patients with LUSC. This model enhances our understanding of the potential biological mechanisms of the DL model and provides new research perspectives for the interpretability of the prediction model.

## Supplementary Data

The Supplementary data can be found online at: www.aginganddisease.org/EN/10.14336/AD.2024.0169.

## Data Availability

The raw data supporting the conclusions of this article will be made available by the authors, without undue reservation.
