# Supplementary material for: Deep Learning Based on Computed Tomography Predicts Response to Chemoimmunotherapy in Lung Squamous Cell Carcinoma
Source: Aging Dis. 2024 May 12;16(3):1674–90. doi: 10.14336/AD.2024.0169 (PMC12096918; doi:10.14336/AD.2024.0169)
Supplement: Supplementary file 1 — The Supplementary data can be found online at: www.aginganddisease.org/EN/10.14336/AD.2024.0169. [file ad-16-3-1674-s.pdf]

SUPPLEMENTARY DATA

Deep Learning Based on Computed Tomography Predicts Response to Chemoimmunotherapy in Lung Squamous Cell Carcinoma

Jie Peng, Baowen Xie, Honglian Ma, Rui Wang, Xiao Hu, Zhongjun Huang

© 2024. Peng J et al. Published online at http://www.aginganddisease.org/EN/10.14336/AD.2024.0169

SUPPLEMENTARY DATA

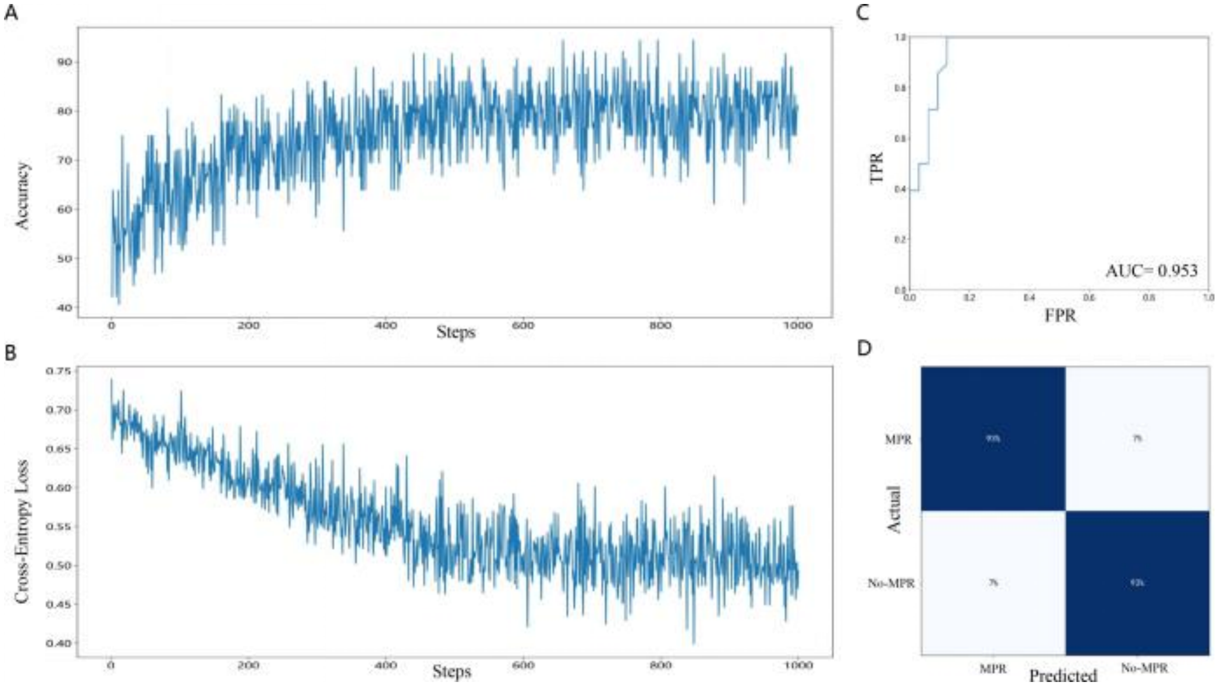

Supplementary Figure 1. Training process and predictive performance of the DL model. (A) Accuracy curves of the training process were presented based on ResNet50. (B) Cross-entropy loss curves of the training process were presented based on ResNet50. (C) DL model showing the AUC in the discovery set. (D) DL model showing the confusion matrix in the discovery set. DL, deep learning; AUC, area under the curve.

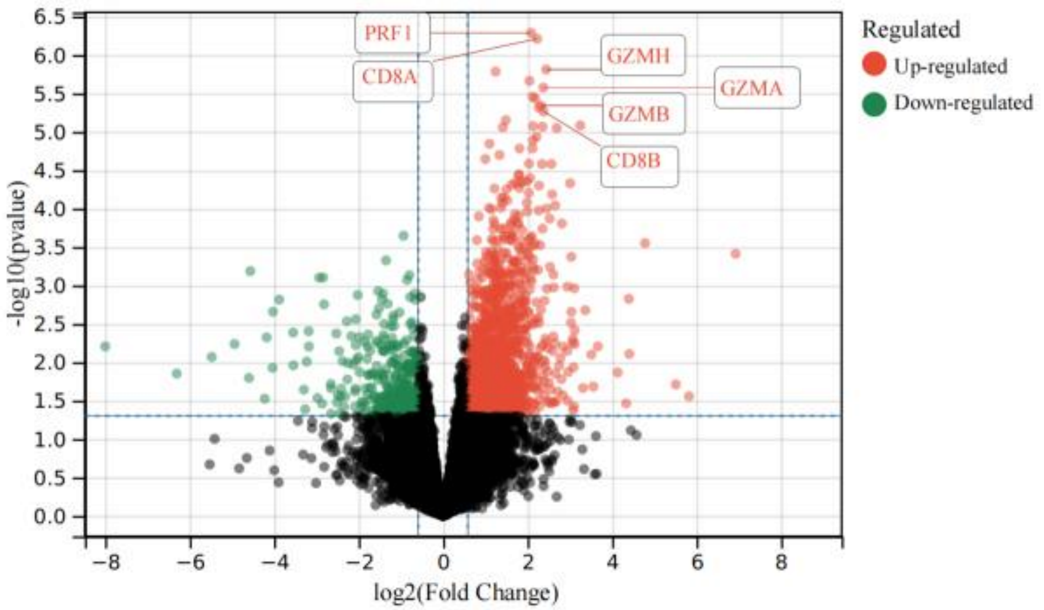

Supplementary Figure 2. Volcano diagram of gene expression profiles compared to P-MPR and P-No-MPR groups. Red and blue dots represent upregulated and downregulated genes, respectively. The x and y axes denote the fold change and statistical significance, respectively. P-MPR; predictive major pathological response.

© 2024. Peng J et al. Published online at http://www.aginganddisease.org/EN/10.14336/AD.2024.0169

SUPPLEMENTARY DATA

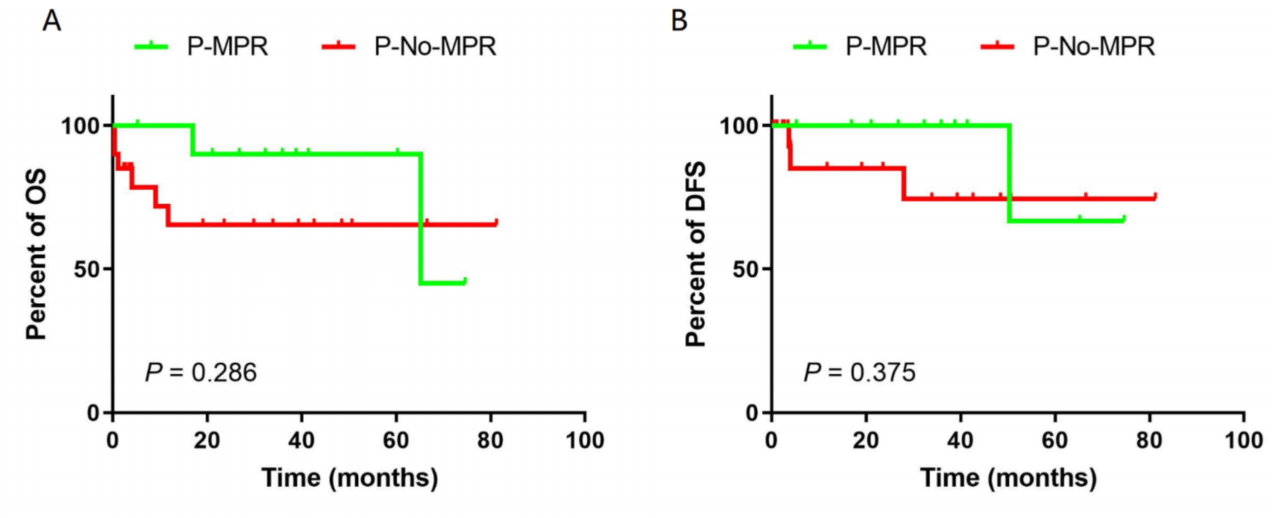

Supplementary Figure 3. Analysis of OS and DFS in the P-MPR and P-No-MPR groups. (A) OS time was compared with P-MPR and P-No-MPR groups. (B) DFS time was compared with P-MPR and P-No-MPR groups. OS, overall survival; DFS, disease-free survival; P-MPR, predictive major pathological response.

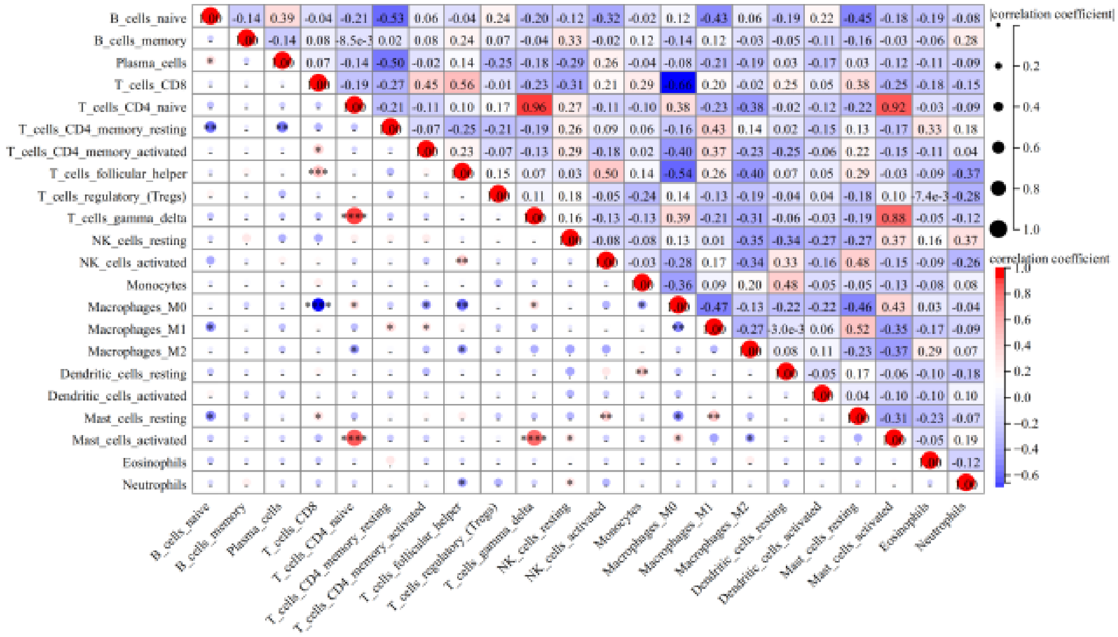

Supplementary Figure 4. Analysis of individual immune cells. Red represents a positive correlation, blue shows a negative correlation, and the circle size is related to the correlation coefficient.

© 2024. Peng J et al. Published online at http://www.aginganddisease.org/EN/10.14336/AD.2024.0169

SUPPLEMENTARY DATA

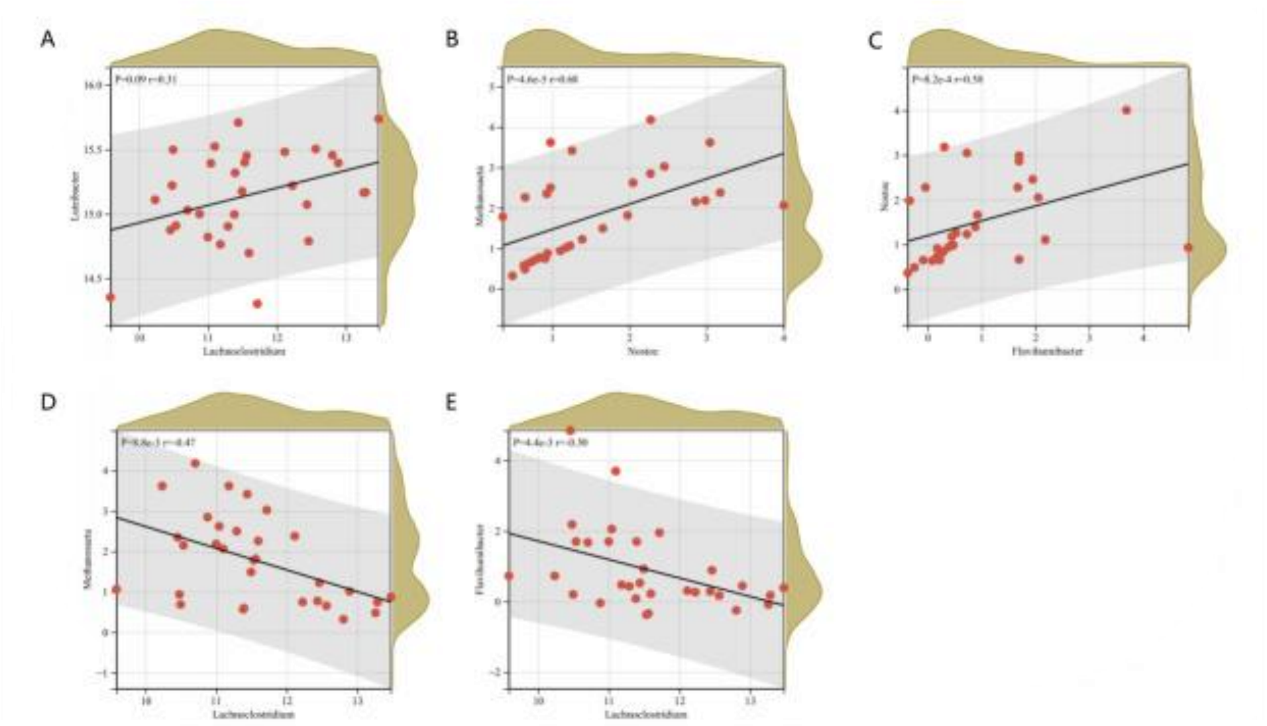

Supplementary Figure 5. Correlations among seven cancer microbiomes. (A) Association between *Luteibacter* and *Lachnoclostridium*. (B) Association between *Methanosaeta* and *Nostoc*. (C) Association between *Nostoc* and *Flavihumibacter*. (D) Association between *Methanosaeta* and *Flavihumibacter*. (E) Association between *Flavihumibacter* and *Lachnoclostridium*.

Supplementary Table 1. Model performance of subgroups in clinical characteristics

**Groups**	**AUC (95% CI)**	**Sensitivity (95% CI)**	**Specificity (95% CI)**	***P* value**
Age (≤ 60 years)	0.97 (0.92–1.00)	90.00 (68.30–98.77)	90.91 (58.72–99.77)	< 0.001*
Age (> 60 years)	0.91 (0.85–0.98)	94.44 (81.34–99.32)	83.33 (68.64–93.03)	< 0.001*
Gender (female)	1.00 (1.00–1.00)	75.00 (30.06–98.72)	100.00 (5.12–10.00)	0.157
Gender (male)	0.92 (0.87–0.97)	92.73 (82.41–97.98)	83.67 (70.34–92.68)	< 0.001*
Smoking status (smoker)	0.93 (0.88–0.98)	93.75 (82.80–98.69)	84.09 (69.93–93.36)	< 0.001*
Smoking status (non-smoker)	0.93 (0.79–1.00)	87.50 (47.35–99.68)	100.00 (66.37–100.00)	0.002*
Stage (II)	0.85 (0.72–0.99)	85.71 (57.19–98.22)	75.00 (47.62–92.73)	< 0.001*
Stage (III)	0.96 (0.92–1.00)	95.24 (83.84–99.42)	91.89 (78.09–98.30)	< 0.001*
Cycles (2)	0.95 (0.91–1.00)	97.37 (86.19–99.93)	87.50 (73.20–95.81)	< 0.001*
Cycles (3–4)	0.86 (0.72–1.00)	83.33 (58.58–96.42)	76.92 (46.19–94.96)	< 0.001*
Radiological response (CR/PR)	0.91 (0.85–1.00)	90.32 (74.25–97.96)	87.76 (75.23–95.37)	< 0.001*
Radiological response (SD/PD)	0.92 (0.77–1.00)	92.00 (73.97–99.02)	75.00 (19.41–99.37)	0.007*

AUC, area under the curve; CI, confidence interval; CR, complete response; PR, partial response; SD, stable disease; PD, progressive disease.

© 2024. Peng J et al. Published online at http://www.aginganddisease.org/EN/10.14336/AD.2024.0169

SUPPLEMENTARY DATA

Supplementary Table 2. Top 100 genes of different expressions between P-MPR and P-No-MPR in the LUSC set.

Tag	logFC	AveExpr	t	*P*.Value	adj.P.Val	*P*
BTN3A1	730.2143	819.239	6.823134777	1.59E-07	0.003261116	-0.694612777
CD8A	581.2675523	391.5104032	5.955696048	1.70E-06	0.011705726	-1.200716068
SLA2	115.1773068	87.03704194	5.867554822	2.17E-06	0.011705726	-1.256300896
CD3G	73.62726045	54.6369871	5.832101575	2.40E-06	0.011705726	-1.278875316
CXCR6	229.280635	164.0460097	5.769463541	2.86E-06	0.011705726	-1.319061943
AKAP5	67.26600818	66.88829355	5.640326558	4.09E-06	0.011975192	-1.40312651
GZMA	413.7820682	261.0766452	5.624218849	4.28E-06	0.011975192	-1.413726311
BTN3A3	501.0794182	545.8377742	5.592432107	4.67E-06	0.011975192	-1.43471771
TMIGD2	12.71709182	8.949506452	5.468789984	6.60E-06	0.013657818	-1.517295926
CD8B	161.2894986	106.6934226	5.457736218	6.81E-06	0.013657818	-1.524749894
GZMH	159.0176359	98.82306774	5.431200256	7.33E-06	0.013657818	-1.542691566
RASGEF1B	256.9136309	254.5913613	5.130910973	1.70E-05	0.028992632	-1.750311584
KLRC4	9.049334091	4.982629032	5.099611031	1.85E-05	0.029212213	-1.772426248
ITGAE	333.3340773	419.4174839	5.0409242	2.18E-05	0.031966972	-1.814124748
LPIN2	405.5565682	657.5264194	4.820437769	4.05E-05	0.052110528	-1.973429881
IL15	122.9533023	104.0196161	4.807709872	4.19E-05	0.052110528	-1.982749362
CST7	178.0859159	165.4579516	4.796760261	4.32E-05	0.052110528	-1.990777211
GZMB	436.7343345	299.4953677	4.76399745	4.74E-05	0.053932122	-2.014854933
CD244	51.85821636	33.94202258	4.69209878	5.79E-05	0.062449661	-2.067989679
NKG7	314.8990359	229.7184226	4.645487689	6.59E-05	0.067563274	-2.102648292
FAM26F	188.104165	157.6766742	4.524129693	9.24E-05	0.079670131	-2.193636648
HLA-F	1617.691859	1647.143742	4.513556917	9.52E-05	0.079670131	-2.201613202
GBP4	1222.058777	1244.14271	4.503714874	9.78E-05	0.079670131	-2.209045396
LCK	297.5080914	275.9209323	4.486391468	0.000102649	0.079670131	-2.222143235
MYO1G	263.3507436	325.1346581	4.476943302	0.00010538	0.079670131	-2.22929537
UBE2L6	2032.04525	2406.453065	4.429738243	0.000120136	0.079670131	-2.265118234
PDCD1	63.48494636	62.94580645	4.42890695	0.000120413	0.079670131	-2.265750399
CCL5	1425.271331	1189.8032	4.423887961	0.000122102	0.079670131	-2.269568089
TAP1	3572.437091	4721.610968	4.418434683	0.000123963	0.079670131	-2.273717971
CD7	254.1192177	235.7493903	4.417131414	0.000124412	0.079670131	-2.274710028
HLA-B	25049.27255	31404.03355	4.399235152	0.000130741	0.079670131	-2.288343814
RGN	44.35254955	29.94040645	4.394068244	0.000132627	0.079670131	-2.292283885
HLA-C	19228.41823	21448.54161	4.392013681	0.000133385	0.079670131	-2.293851081
PRF1	562.3570541	371.5049065	4.39027019	0.000134031	0.079670131	-2.295181204
APOL3	504.3894895	518.6548806	4.384913632	0.000136035	0.079670131	-2.299268962
TNFSF13B	109.9234495	102.7364	4.351704506	0.000149141	0.084712969	-2.324651849
IL2RB	540.0487	536.6554839	4.34268494	0.000152911	0.084712969	-2.331557542
CD226	24.59927227	27.62462903	4.328339329	0.000159103	0.084789251	-2.342551193
APOL6	1307.744659	1881.374677	4.323331599	0.000161322	0.084789251	-2.34639174
IL9R	12.90768182	9.159545161	4.299254692	0.000172426	0.086431379	-2.364877659
GPR174	18.39115409	13.88254839	4.280177507	0.000181757	0.086431379	-2.379548913
LAP3	941.4202864	2087.35571	4.275705458	0.000184016	0.086431379	-2.38299116
IL18RAP	32.85527318	27.09446774	4.269453538	0.00018722	0.086431379	-2.387805329
UBD	2762.14674	1631.52939	4.265676296	0.000189182	0.086431379	-2.390714995
KLRK1	104.6887845	82.78270323	4.264598664	0.000189746	0.086431379	-2.391545259

© 2024. Peng J et al. Published online at http://www.aginganddisease.org/EN/10.14336/AD.2024.0169

CD247	129.7179545	131.4514516	4.254669497	0.000195017	0.086901401	-2.399198283
HCP5	1173.101527	1164.602032	4.22738251	0.00021026	0.091700053	-2.420258244
HLA-A	16869.82591	25887.83226	4.211343508	0.000219761	0.091789752	-2.432656005
TAPBPL	421.2903273	666.7005161	4.203840786	0.00022435	0.091789752	-2.438460154
PTPRC	1044.117115	1190.7396	4.19953579	0.000227026	0.091789752	-2.441791869
FGL2	525.7809473	592.2672129	4.194247139	0.000230355	0.091789752	-2.445886189
RERG	304.8936259	195.6847258	4.190326286	0.000232855	0.091789752	-2.448922544
KLRC1	27.62254318	15.14762258	4.148974268	0.000260903	0.095816266	-2.48099411
SAMHD1	627.0298682	928.7844194	4.148688564	0.000261108	0.095816266	-2.481215994
PTPN22	101.7153845	107.3514258	4.147089799	0.000262257	0.095816266	-2.482457708
NCF1	116.9369236	158.7171871	4.141018521	0.000266668	0.095816266	-2.487174242
ABI3	155.8137809	223.897071	4.130712426	0.000274324	0.095816266	-2.495184772
GIMAP6	270.2838282	319.3991548	4.129450538	0.000275276	0.095816266	-2.496165944
CD2	358.23211	336.0415097	4.128770563	0.000275791	0.095816266	-2.496694685
CRTAM	37.68427364	33.78032903	4.076307165	0.000318468	0.10879915	-2.537555515
CASS4	58.25063091	66.23543871	4.067360561	0.000326367	0.109143521	-2.544536135
FASLG	38.40395273	27.68232903	4.058443485	0.000334432	0.109143521	-2.551497251
CCL21	1154.207349	875.7060065	4.038362889	0.000353317	0.109143521	-2.56718584
GNLY	316.7427818	199.6679	4.037163689	0.000354478	0.109143521	-2.568123299
RSPO2	18.64153455	9.568358065	4.036502287	0.000355119	0.109143521	-2.568640368
HLA-DRB6	328.8212168	345.0554613	4.030312999	0.000361179	0.109143521	-2.573479901
DOK2	192.3119191	235.6057032	4.029941821	0.000361545	0.109143521	-2.573770185
FGFR1OP2	214.6290636	586.891	4.029408997	0.000362072	0.109143521	-2.574186895
ITGB7	218.6989564	297.7555355	4.014882865	0.000376731	0.111916482	-2.585551998
TNFSF8	37.18226136	41.15758065	3.997379175	0.00039517	0.114209488	-2.599258141
ADORA2A	96.62059409	140.7520419	3.996987197	0.000395593	0.114209488	-2.599565218
MMAA	91.34702227	193.9761645	3.9894138	0.000403852	0.114974469	-2.605499416
HLA-E	6101.663955	10501.15806	3.975105016	0.00041992	0.11791129	-2.616717274
B2M	55396.66636	55533.45161	3.969729918	0.000426117	0.118034356	-2.62093329
HLA-DPA1	4443.582609	5117.413419	3.956481863	0.000441775	0.119104309	-2.631329169
PRG2	9.397672727	8.124358065	3.954827116	0.00044377	0.119104309	-2.632628122
IFIH1	409.8230455	668.1425806	3.949970653	0.000449676	0.119104309	-2.636440954
SAP30	94.92571182	176.0788871	3.947085504	0.000453222	0.119104309	-2.638706504
ARID5A	251.98815	621.386	3.926966607	0.000478716	0.123589124	-2.654513023
KLRD1	75.34764182	50.2175	3.924188015	0.000482346	0.123589124	-2.656697154
ALPK1	260.2123364	411.1547097	3.919419634	0.000488638	0.12365558	-2.660445989
CXCR3	72.20285364	72.80497742	3.897188283	0.00051905	0.125580429	-2.677934058
CCR5	176.4071568	196.7781129	3.893875475	0.000523737	0.125580429	-2.680541435
SLC16A12	9.107530455	7.346319355	3.893752454	0.000523912	0.125580429	-2.680638267
HLA-DPB1	3159.231691	3547.315097	3.892894072	0.000525134	0.125580429	-2.681313926
GIMAP4	319.9910159	426.2122742	3.888319905	0.000531692	0.125580429	-2.684914792
TIFA	234.4540182	393.5201613	3.887411835	0.000533003	0.125580429	-2.685629721
TRIM22	1047.827632	1221.067355	3.870757224	0.000557623	0.128601026	-2.698746555
ABCD2	22.05848909	20.09952581	3.868631297	0.000560845	0.128601026	-2.700421507
CD160	8.100472727	9.47386129	3.866139236	0.000564645	0.128601026	-2.702385098
BTN2A2	190.6513773	328.6728387	3.85221326	0.000586347	0.130064128	-2.713361317
CHI3L1	1505.318688	1950.628471	3.850699396	0.000588755	0.130064128	-2.714554862
PSMB9	906.9146636	937.094129	3.849853241	0.000590105	0.130064128	-2.715222007

© 2024. Peng J et al. Published online at http://www.aginganddisease.org/EN/10.14336/AD.2024.0169

IL12RB1	74.38605136	77.77803548	3.839339564	0.000607133	0.132393787	-2.723513152
ELF2	189.2063864	613.865	3.831589286	0.000619993	0.133774841	-2.729627052
NMI	390.6273182	600.3631935	3.825689018	0.000629961	0.134509734	-2.734282655
ID2	513.9006773	754.9819032	3.790158382	0.000693382	0.145647502	-2.762337329
LYZ	8080.4822	6931.415677	3.787071092	0.000699179	0.145647502	-2.764776514
CD96	196.0150918	207.5418323	3.782588323	0.000707679	0.145647502	-2.768318635
PCYOX1	-1263.879518	2522.510323	-3.778964594	0.000714625	0.145647502	-2.77118232

P-MPR, predictive major pathological response; LUSC, lung squamous cell carcinoma.

Supplementary Table 3. Risk factors for P-MPR in TCGA-LUSC set.

Intercept and variables	β	OR (95% CI)	*P*-value
Intercept	4.821890e+04	8.97 (0.00–3.01e+13)	0.229
Ulvibacter	6.239299e-01	0.63 (0.15–2.18)	0.457
Lachnoclostridium	2.042141e-01	0.87 (0.02–0.89)	0.068
Demetria	1.794143e+01	1.58 (1.51–93.78)	0.069
Flavihumibacter	2.906401e+00	1.10 (0.53–46.21)	0.334

OR, odds ratio; CI, confidence interval; P-MPR, predictive major pathological response; TCGA, The Cancer Genome Atlas; LUSC, lung squamous cell carcinoma. **P*-value < 0.05.

© 2024. Peng J et al. Published online at http://www.aginganddisease.org/EN/10.14336/AD.2024.0169
